# Up-Regulation of MicroRNA-190b Plays a Role for Decreased IGF-1 That Induces Insulin Resistance in Human Hepatocellular Carcinoma

**DOI:** 10.1371/journal.pone.0089446

**Published:** 2014-02-20

**Authors:** Tzu-Min Hung, Cheng-Maw Ho, Yen-Chun Liu, Jia-Ling Lee, Yow-Rong Liao, Yao-Ming Wu, Ming-Chih Ho, Chien-Hung Chen, Hong-Shiee Lai, Po-Huang Lee

**Affiliations:** 1 Department of Surgery, National Taiwan University Hospital and National Taiwan University College of Medicine, Taipei, Taiwan; 2 Department of Medical Research, E-DA Hospital, Kaohsiung, Taiwan; 3 Graduate Institute of Clinical Medicine, College of Medicine, National Taiwan University, Taipei, Taiwan; 4 Department of Internal Medicine, National Taiwan University Hospital and National Taiwan University College of Medicine, Taipei, Taiwan; 5 Department of Surgery, E-DA Hospital, Kaohsiung, Taiwan; University of Hong Kong, Hong Kong

## Abstract

**Background & Aims:**

Insulin-like growth factor, (IGF)-1, is produced mainly by the liver and plays important roles in promoting growth and regulating metabolism. Previous study reported that development of hepatocellular carcinoma (HCC) was accompanied by a significant reduction in serum IGF-1 levels. Here, we hypothesized that dysregulation of microRNAs (miRNA) in HCC can modulate IGF-1 expression post-transcriptionally.

**Methods:**

The miRNAs expression profiles in a dataset of 29 HCC patients were examined using illumina BeadArray. Specific miRNA (miR)-190b, which was significantly up-regulated in HCC tumor tissues when compared with paired non-tumor tissues, was among those predicted to interact with 3′-untranslated region (UTR) of *IGF-1.* In order to explore the regulatory effects of miR-190b on IGF-1 expression, luciferase reporter assay, quantitative real-time PCR, western blotting and immunofluorecence analysis were performed in HCC cells.

**Results:**

Overexpression of miR-190b in Huh7 cells attenuated the expression of IGF-1, whereas inhibition of miR-190b resulted in up-regulation of IGF-1. Restoration of IGF-1 expression reversed miR-190b-mediated impaired insulin signaling in Huh7 cells, supporting that IGF-1 was a direct and functional target of miR-190b. Additionally, low serum IGF-1 level was associated with insulin resistance and poor overall survival in HCC patients.

**Conclusions:**

Increased expression of miR-190 may cause decreased IGF-1 in HCC development. Insulin resistance appears to be a part of the physiopathologic significance of decreased IGF-1 levels in HCC progression. This study provides a novel miRNA-mediated regulatory mechanism for controlling IGF-1 expression in HCC and elucidates the biological relevance of this interaction in HCC.

## Introduction

IGF-1 is a potent mitogenic factor that exerts anti-apoptotic effects on many cell systems. Experimental and epidemiologic evidence indicate that the IGF axis plays an important role in carcinogenesis [1], [2]. High serum IGF-1 levels are associated with an increased risk for prostate, breast, colon, and lung cancers [2]. As a circulating peptide hormone, IGF-1 is also an important factor in regulating metabolism. IGF-1 has structural homology with insulin and exerts insulin-like effects on glucose and lipid metabolism [3]. Cross-sectional studies suggest that IGF-1 levels may be reduced in insulin resistance and type 2 diabetes [4]. Altered IGF-1 levels have also been implicated in cardiovascular disease, with compelling data suggesting that reduced IGF-1 levels are associated with increased cardiovascular risk [5], [6]. In animal models, mice become insulin resistant when liver synthesis of IGF-1 is deleted [7], [8], and IGF-1 administration corrects this insulin resistance [8].

Since alterations of IGF levels have been implicated in the pathogenesis of several diseases, understanding the mechanisms controlling IGF-1 expression are of great interest. Post-transcriptional regulatory mechanisms, some of which involve microRNAs (miRNAs), have been reported to influence IGF-1 expression [9]. Among neoplasm with activated IGF signaling, some miRNAs acted as tumor suppressors to inhibit the expression of IGF-1 receptor (IGF-1R), such as miR-145, and miR-99a in breast cancer and HCC, respectively [10], [11]. Additionally, miR-29 acts as an antifibrogenic mediator by interfering with profibrogenic cell communication via IGF-1 [12]. Furthermore, miR-1 and miR-206 have been shown to target the 3′-untranslated region (3′-UTR) of IGF-I mRNA and reduce IGF-1 expression; miR-1 levels increased in rat cardiomyoctes during glucose-induced apoptosis and antagonized the anti-apoptotic action of IGF-1 [13], [14]. IGF-I was downregulated by miR-320 in myocardial microvascular endothelial cells of type 2 diabetic rats. Expression of miR-320 was up-regulated in the diabetic rat model, impairing angiogenesis by repressing IGF-I expression [15].

Unlike the positive correlation between IGF-1 levels and other cancers, a reduced serum level of IGF-1 has been documented in hepatocellular carcinoma (HCC) [16], [17]. In patients with hepatitis C virus–related cirrhosis, HCC development was accompanied by significant reduction in serum IGF-1 levels [18]. Serum IGF-1 levels were significantly decreased in HCC patients compared to healthy subjects [19]. Since most serum IGF-1 originates in the liver, destruction of the liver parenchyma would reduces serum IGF-1 levels [20]. However, in HCC, upstream regulators that may lead to this decrease are not well defined. Furthermore, the physiopathological significance of decreased IGF-1 levels in the progression of HCC remains unclear. In the present study, we hypothesized that dysregulation of miRNAs in HCC may modulate IGF-1 expression post-transcriptionally. In addition, we demonstrated that IGF-1 plays a role in insulin resistance other than the expected reduction in its own levels in HCC.

## Materials and Methods

### Ethics Statement

This study was approved by the Research Ethics Committee of the National Taiwan University Hospital, Taipei, Taiwan. Written informed consent was obtained from all included subjects. Another cohort of 29 HCC patients, which tissues were used to conduct the gene expression analysis, was registered at ClinicalTrials.gov (NCT01247506) as described in our previous study [21].

### Subjects

This study collected preoperative serum samples from 102 HCC patients who underwent curative resection between January 2004 and December 2005 at National Taiwan University Hospital and were followed until December 2011. Patients’ clinical and pathological data were retrieved from medical records. Deaths of enrolled patients were confirmed using the mortality data bank of Taiwan Cancer Registry. Two control groups were included. Serum from healthy subjects was obtained from living liver donors at the same hospital. A second “viral hepatitis” group included chronic hepatitis B virus or hepatitis C virus patients without HCC.

### Enzyme-linked Immunosorbent Assay (ELISA)

Serum IGF-1 levels of all subjects were measured using a commercial enzyme-linked immunosorbent assay (ELISA) kit (R&D Systems, Minneapolis, MN, USA). Basal serum insulin was measured using a commercially available insulin ELISA kit (Mercodia, Uppsala, Sweden). ELISA assays were performed according to the manufacturer’s instructions. The index of insulin resistance (IR) was calculated using the homeostasis model of assessment (HOMA) formula: fasting plasma glucose (mg/dL) × fasting serum insulin (mU/L)/405.

### Isolation of Total RNA, miRNA Microarray and Quantitative Real-time PCR Analysis of miRNA and mRNA

Total RNA from human HCC samples and cancer cell lines were extracted using Trizol reagent (Invitrogen, Carlsbad, CA, USA). 29 HCC tumor tissues (T) and their paired nontumor tissues (NT) were selected for microRNA profiling. Total RNA was analyzed by illumina BeadArray (human v2 miRNA panel) according to the manufacturer’s instructions. miRNAs with false discovery rate (FDR) <0.05 using paired t-test and T/NT ratio >2 or <0.5 were identified as differentially expressed genes. For miRNA analyses by quantitative real-time polymerase chain reaction (qRT-PCR), 100 ng of total RNA was reverse-transcribed using the TaqMan miRNA Reverse Transcription Kit (Applied Biosystems, Carlsbad, CA, USA). The expression levels of mature miR-190b and control RNU6B were determined by qRT-PCR with the TaqMan Universal PCR Master Mix in StepOne Real-time PCR System (Applied Biosystems). TaqMan probes from Applied Biosystems were used to assess the expression of miR-190b (ID 002263) and RNU6B (ID 001093). For mRNA expression detection, one microgram total RNA was reverse-transcribed using random hexamer and MMLV reverse transcriptase (Fermentas, Glen Burnie, MD, USA). qRT-PCR was subsequently performed using TaqMan Gene Expression Assays (Applied Biosystems). The assay ID numbers of the validated genes are as follows: Hs01555481 for IGF-1 and Hs99999905 for GAPDH. miRNA and mRNA transcript levels were normalized to RNU6B and GAPDH mRNA levels (ΔCT), respectively.

### Expression Plasmids

The EGFP-tagged pEZX-MR04 vectors expressing precursor miR-190b and scrambled sequences [named pre-miR-190b and pre-negative control (NC)], the mCherry-tagged pEZX-AM01 vectors expressing the miR-190b inhibitor and scrambled sequences (named anti-miR-190b and anti-NC), as well as luciferase vectors (pEZX-MT01) containing IGF-1 3′UTRs, were obtained from GeneCopoeia, Rockville MD. Human TrueClone IGF-1 plasmid was purchased from OriGene Technologies (Rockville, MD). The cloning expression vector is pCMV6-XL.

### Luciferase Reporter Assay

HEK293T cells (1*10^5^) were seeded in 24-well plates the day before transfection. Luciferase vectors and precursor control or precursor-miR-190b or inhibitor control or inhibitor miR-190b were co-transfected into cells using TurboFect reagent (Fermentas, Glen Burnie, MD, USA) following the manufacturer’s protocol. Forty-eight hours post-transfection, cell extracts was assayed for luciferase activity using the Luc-Pair miR luciferase assay kit (Genecoepia, Rockville, MD). Relative luciferase activities were expressed as luminescence units normalized to Renilla luciferase activity.

### miRNA Transfection and Establishment of Stable Cell Clones

The human HCC cell lines, Huh7 and HepG2, were maintained at 37°C with 5% CO2 in Dulbecco’s modified Eagle’s medium (Invitrogen, Carlsbad, CA) supplemented with 10% heat-inactivated fetal bovine serum and 100 U of penicillin and 100 µg of streptomycin/ml. Stable cell clones were established by transfecting plasmids (as described above) into Huh7 cells using TurboFect reagent. Stable cell clones were generated with 2 µg/ml and maintained in culture with 1 µg/ml of puromycin (Invitrogen, Carlsbad, CA, USA). These subclones were named Huh7-Pre-NC-1, Huh7-Pre-miR190b-3, Huh7-Pre-miR190b-6, Huh7-Anti-NC-1, Huh7-Anti-miR190b-2 and Huh7-Anti-miR190b-5.

### Western Blotting

Whole cell lysate was subjected to 12.5% sodium dodecyl sulfate (SDS)–polyacrylamide electrophoresis gel, transferred to polyvinylidene difluoride membrane, blotted with rabbit polyclonal IGF-1 antibody (Santa Cruz Biotechnology, Santa Cruz, CA, USA) and followed by the secondary antibody. The enhanced chemiluminescence detection system was used to detect the immunocomplex. Antibodies directed against IRS1, FOXO1, GSK3β, and phosphor-specific antibodies directed against p-IRS1 (Ser612 or 318), p-FOXO1 (Ser256) and p-GSK3β (Ser9) were supplied by Cell Signaling Technology (Danvers, MA, USA). Anti-β-actin antibody was purchased from Novus (Littleton, CO, USA).

### Immunofluorescence

Cells were plated onto glass cover slips and fixed in 4% (vol/vol) paraformaldehyde/phosphate-buffered saline (PBS). They were subsequently permeabilized in PBS containing 0.5% (vol/vol) Triton X-100 for 15 min and were blocked in PBS containing 5% (wt/vol) bovine serum albumin for an hour. Cells were labeled with rabbit anti-IGF-1 or anti-FOXO1 antibody, followed by AF-488-conjugated goat anti-rabbit immunoglobulin G (Molecular Probes, Carlsbad, CA). Nucleus was stained with Hoechst 33258. Photographs were taken using a fluorescence microscope (Axio Imager A1, ZEISS).

### Glucose Production Assay

Glucose production was measured using a commercial kit (Glucose Colorimetric Assay Kit II, BioVision, Milpitas,CA). Briefly, Huh7 stable cells were washed with PBS and incubated with glucose production buffer consisting of glucose-free Dulbecco’s modified Eagle’s medium (Invitrogen, Carlsbad, CA, USA), without phenol red, supplemented with a gluconeogenic substrate (2 mM sodium pyruvate and 20 mM sodium lactate). After incubation for 4 hours, the medium was collected, and the total glucose concentration was measured.

### Statistical Analysis

All statistical analyses were performed using SPSS 16.0 statistical software package (SPSS, Chicago, IL, USA). Data were presented as mean ± SD. Data were analyzed using the Wilcoxon signed rank test for comparison of paired data, the One-way ANOVA for more than two groups and the Student’s t-test for unpaired comparison. Pearson correlations were used to determine correlation coefficients. Kaplan-Meier method was used for survival analysis, and log-rank test was used to compare differences. *P*<0.05 was considered statistically significant.

## Results

### Circulating and Hepatic Levels of IGF-1 were Decreased in Patients with HCC

The circulating levels of IGF-1 were analyzed in the cohort of 102 HCC patients. When compared to patients with viral hepatitis and healthy subjects, serum IGF-1 levels were significantly decreased in patients with HCC (Table 1). Besides, HCC and viral hepatitis groups showed significantly higher levels of AST, ALT and bilirubin than those of healthy controls (Table 1), which supported that destruction of the liver parenchyma reduced serum IGF-1 levels. However, compared to the viral hepatitis group, the HCC group showed no significant differences in AST levels (*P* = 1.000) and even had significantly lower levels of ALT (*P = *0.022) and bilirubin (*P* = 0.019). Liver damage alone seems not to explain the greater decreases in IGF-1 levels of HCC patients compared with those of the viral hepatitis group.

**Table 1:** Demographic data and clinical features of healthy control, viral hepatitis and hepatocellular carcinoma groups.

Variablemean ± SD	Healthy control^1^(n = 38)	Viral hepatitis^2^(n = 50)	HCC^3^(n = 102)	*P* vale(1 vs.2)	*P* vale(1 vs.3)	*P* vale(2 vs.3)
Sex (Male/Female)	19/19	30/20	77/25			
Age (years)	33.6±11	56.4±4.2	58.1±11.6	<0.001	<0.001	0.970
Serum IGF-1 level (ng/mL)	106.3±46.7	70.0±29.1	46.4±13.9	<0.001	<0.001	<0.001
AST (U/L)	17.8±5.6	61.4±49.2	58.3±40.6	<0.001	<0.001	1.000
ALT (U/L)	16.5±10.3	96.3±112.2	63.6±52.5	<0.001	<0.01	0.022
Bilirubin (mg/dL)	0.7±0.3	1.1±0.4	0.9±0.4	<0.001	<0.05	0.019

The significance of the difference between the three groups was tested first using an analysis of variance (ANOVA); then, if proven significant, the Bonferroni test was used to assess the individual differences between each pair of groups.

n = number of subjects.

AST, aspartate aminotransferase; ALT, alanine aminotransferase.

**Figure 1:**
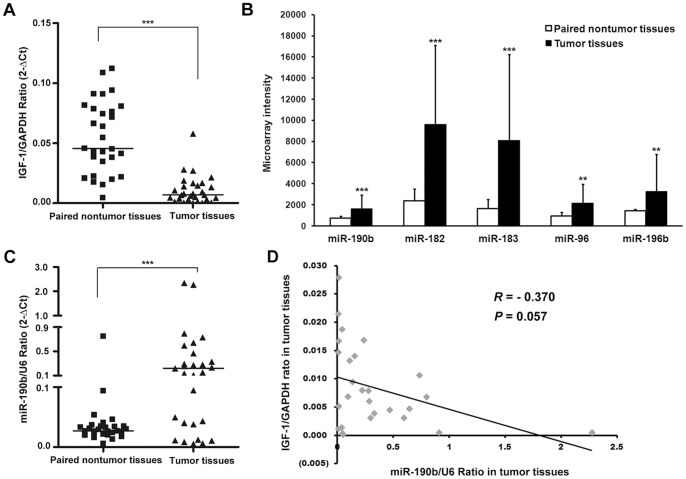
Identification of miRNA that targets IGF-1. (A) qRT-PCR analysis showing that IGF-1 expression levels were significantly down-regulated in HCC tumor tissues as compared with paired nontumor tissues. The horizontal line indicates the median. The expression level of IGF-1 was normalized by GAPDH. (B) An integrative analysis of miRNA BeadArray with TargetScan predictions defined 5 miRNAs that are up-regulated in HCC tumor tissues and could potentially bind to the 3′-UTR of IGF-1 mRNA. (C) qRT-PCR analysis of miR-190b expression in HCC tumor and nontumor tissues. The horizontal line indicates the median. The expression level of miR-190b was normalized by U6. (D) Correlation between expression levels of IGF-1 and miR-190b of tumor tissues in 29 HCC patients. Linear regression coefficients and statistical significance are indicated. ***P<0.001; **P<0.01.

The levels of hepatic IGF-1 were analyzed in a further cohort of 29 HCC patients from whom tumor and adjacent non-tumor tissues were collected. As shown in Figure 1A, using qRT-PCR demonstrated that IGF-1 expression was downregulated >2-fold in 90% (26/29) of tumor tissues examined. The relative expression of IGF-1 was significantly lower in tumor tissues as compared to paired non-tumor tissues (*P*<0.001; Figure 1A). This result raises a possibility that tumor-specific factors might also contribute to the decline of IGF-1 in HCC.

### Identification of miRNA that Targets IGF-1

Determination of miRNA expression profiles of 29 HCC tumor tissues and their paired non-tumor tissues identified 16 miRNAs that were significantly and differentially expressed between HCC tumor tissues and their paired non-tumor tissues, including 8 up-regulated and 8 down-regulated (Table S1). Since miRNAs act as negative regulators, up-regulated miRNAs resulted in down-regulated target mRNAs, 8 up-regulated miRs were chosen for further *in silico* analysis. Using TargetScan Human V5.1 prediction, 5 miRNAs that potentially bind to the 3′ UTR of IGF-1 mRNA were selected (Figure 1B). To validate the reliability of the microarray test, miR-190b, one of the most statistically significant miR, was selected for qRT-PCR examination. The expression of miR-190b was significantly up-regulated in 66% (19/29) of HCC samples compared with the paired nontumor tissues (Figure 1C). Next, we correlated the expression of IGF-1 to that of miR-190b in the 29 HCC tumor tissues and identified a marginally reverse correlation (*P* = 0.057; Figure 1D), implying that miR-190b dysregulation might be associated with the decline of IGF-1.

### IGF-1 is a Direct Target of miR-190b

**Figure 2:**
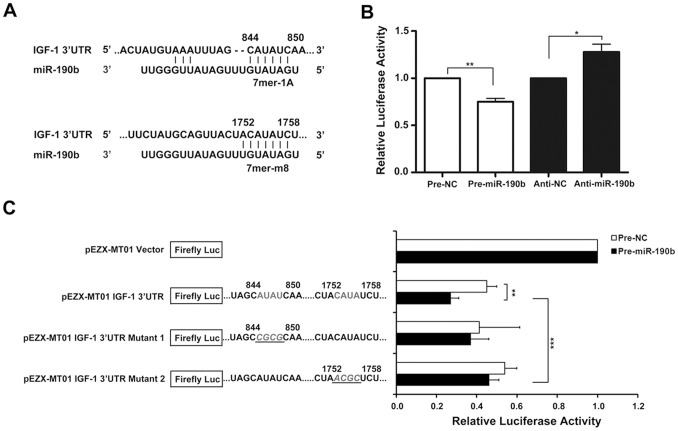
IGF-1 was a direct target of miR-190b. (A) Base pairing complement suggests two putative miR-190b binding positions at 844–850 and 1752–1758 of the IGF-1 3′UTR. (B) The effect of precursor miR-190b or miR-190b inhibitor on pEZX-IGF-1 3′UTR luciferase activity. Luciferase assays were done on HEK293 T cells. Significant differences were compared to each negative control (NC). (C) HEK293T cells were transiently transfected with pre-miR-190b or pre-NC vector together with the pEZX-MT01 empty vector, a modified pEZX-MT01 vector containing wild-type IGF-1 3′UTR, or two mutant IGF-1 3′UTR carrying mutations for each putative miR-190b binding site. Luciferase activity of pEZX-MT01 empty plasmid was set to 1. ***, *P*<0.001; **, *P*<0.01; *, *P*<0.05.

Analysis of the 3′UTR sequence of IGF-1 using TargetScan revealed two possible binding sites for miR-190b at positions 844–850 and 1752–1758 (Figure 2A). To determine whether miR-190b affects IGF-1 expression through these putative elements, a luciferase reporter construct carrying these elements was employed. Precursor miR-190b transfection significantly decreased luciferase activities whereas its inhibitor transfection increased them (Figure 2B). Inclusion of the *IGF-1* 3′ UTR into a luciferase reporter construct reduced luciferase activity compared to a reporter lacking *IGF-1* 3′ UTR (Figure 2C), suggesting that this 3′UTR of IGF-1 was inhibited by the endogenous expression of miRs. Overexpression of miR-190b led to further reduction of luciferase activity when the reporter construct contained *IGF-1* 3′UTR (Figure 2C). Two reporter constructs were generated carrying mutations for each predicted miR-190b binding site. As shown in Figure 2C, the suppressive effect of miR-190b was significantly reversed on mutation of binding site 2 (7mer-m8), whereas no significant changes were noted on mutation of binding site 1 (7mer-A1). Taken together, these results revealed that IGF-1 is a direct target of miR-190b.

### The Regulatory Effects of miR-190b on IGF-1 Expression

**Figure 3:**
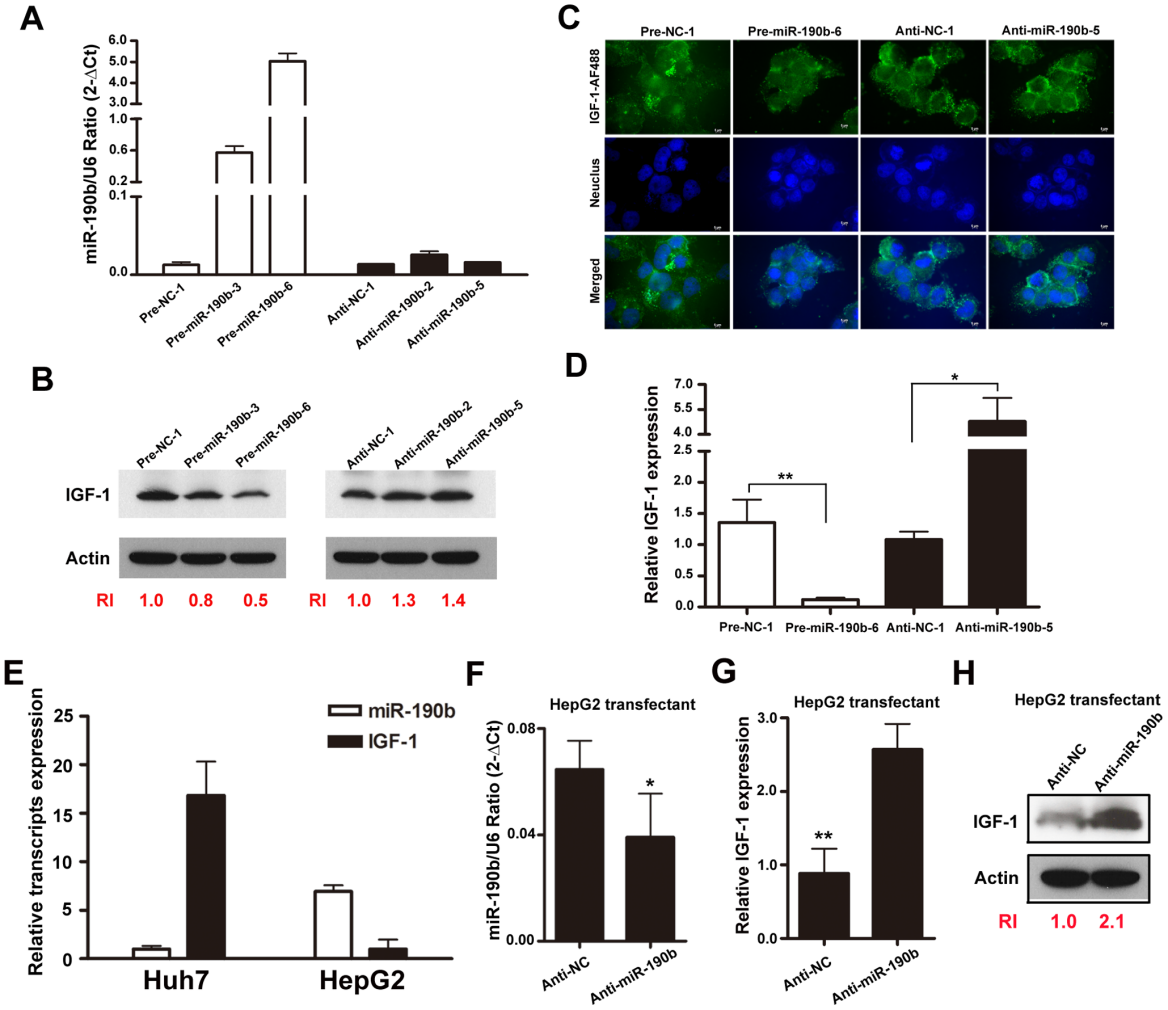
The regulatory effects of miR-190b on IGF-1 expression. (A) qRT-PCR assay verified the relative miR-190b expression in Huh7 stable cells clones. (B&C) Overexpression of miR-190b attenuated IGF-1 protein expression, and blocking miR-190b moderately increased IGF-1 protein expression. These results were assayed by western blotting (B) and immunofluorescence staining (C), respectively. (D) IGF-1 mRNA was detected by qRT-PCR in stable cells expressing precursor miR-190b or inhibitor miR-190b. Relative expression of IGF-1 compared to negative control (NC) was calculated using the 2^−ΔΔCT^ methods. (E) The endogenous transcript levels of miR-190b and IGF-1 were dertermined by qRT-PCR in Huh7 and HepG2 cells. (F&G) qRT-PCR analysis of miR-190b (F) and IGF-1 (G) in HepG2 cells transfected with anti-NC or anti-miR-190b vectors. (H) Western blotting of IGF-1 in HepG2 cells transfected with anti-NC or anti-miR-190b vectors. Relative intensity (RI) shown was calculated by normalization of the intensities of IGF-1 from the internal controls. **, *P*<0.01 and *, *P*<0.05 as compared to each of the negative control cells.

To learn whether miR-190b can affect endogenous IGF-1 protein levels, we established Huh7 stable cells that constitutively express precursor negative control, precursor miR-190b, inhibitor negative control or miR-190b inhibitor. Results of qRT-PCR confirmed that clones expressing precursor miR-190b exhibited greatly increased miR-190b expression when compared to negative controls (Figure 3A, left). However, the principle of our inhibitor is to de-repress microRNA targets [22], so the transfected inhibitors would not alter the amount of endogenous miR-190b (Figure 3A, right). Next, when exploring the corresponding IGF-1 protein levels in these stable clones, western blotting showed dose-dependent reduced expression of IGF-1 in Huh7 stable cells expressing precursor miR-190b (Figure 3B, left). Conversely, increased expression of IGF-1 was observed in stable cells expressing miR-190b inhibitor (Figure 3B, right).

Expression of IGF-1 proteins was also verified by immunofluorescence. Among the miR-190b stable cells, analysis of two representative subclones, namely Huh7-Pre-miR190b-6 and Huh7-Anti-miR190b-5, revealed an attenuated IGF-1 signal in Huh7-Pre-miR190b-6 cells and a moderately elevated IGF-1 signal in Huh7-Anti-miR190b-5 (Figure 3C). Because inhibition of expression by miRNA may also be mediated by mRNA degradation, we examined whether IGF-1 mRNA levels might be affected by miR-190b; consistent overexpression of miR-190b decreased IGF-1 mRNA levels, whereas inhibition of miR-190b increased the levels (Figure 3D), indicating that miR-190b might act to destabilize or degrade IGF-1 mRNA transcripts. To further confirm this, we analyzed the turnover of IGF-1 mRNA in miR-190b overexpressed and control cells. IGF-1 mRNA stability was determined by qRT-PCR after treatment with actinomycin D to inhibit de novo RNA synthesis. As shown in Figure S1, overexpression of miR-190b resulted in a significant decrease in IGF-1 mRNA levels after 6 hr with actinomycin D, on the contrary, IGF-1 mRNA was stable in control cells.

Transient transfection was also performed to clarify that IGF-1 repression was not due to clonal effects of miR-190b stable cells. Analysis of IGF-1 expression confirmed decreased IGF-1 protein levels in cells transfected with precursor miR-190b as compared to precursor controls (Figure S2-A and B). Conversely, IGF-1 expression in cells transfected with miR-190b inhibitor was increased when compared to inhibitor controls (Figure S2-A and B). Immunofluorescence analysis was used to further confirm the relationship between miR-190b and IGF-1. Representative staining is shown in Figure S2-C and D. IGF-1 expression was suppressed in cells transfected with precursor miR-190b (Figure S2-D, indicated by arrows). Cells transfected with empty vector showed no change in IGF-1 expression (Figure S2-C). Collectively, these results indicate that *IGF-1* gene expression is directly and post-transcriptionally suppressed by miR-190b.

Because the endogenous levels of miR-190b were marginally expressed in Huh7 cells, the other cell was used to conduct the loss-of-function analysis of miR-190b again. We transfected the miR-190b inhibitors into HepG2 cells, which have a relatively higher basal levels of miR-190b and lower levels of IGF-1 compared to those of Huh7 cells (Figure 3E). We expected that our miRNAs inhibitor should function more by entrapping than by degradation of the target mature miRNA. But qRT-PCR showed a modest decrease in miR-190b after transfection of the miR-190b inhibitor (Figure 3F) [22]. Furthermore, inhibition of miR-190b in HepG2 cells led to a clear increase of IGF-1 expression both in the mRNA (Figure 3G) and protein levels (Figure 3H). Taken together, these data further confirm that miR-190b can regulate IGF-1 expression.

### Low IGF-1 is Associated with Insulin Resistance and Poor Prognosis in Patients with HCC

**Figure 4:**
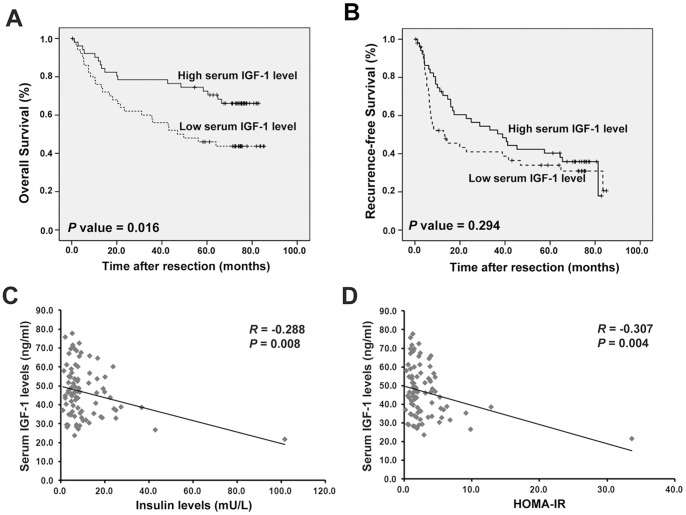
Low IGF-1 is associated with insulin resistance and poor prognosis in HCC patients. (A, B) Kaplan-Meier curves for overall survival (A) and recurrence-free survival (B) in patients with HCC after curative resection. Compared with patients with high IGF-1 levels, patients with low IGF-1 levels exhibited significantly shorter overall survival but no significant difference in recurrence-free survival. Statistical significance was calculated using the log-rank test. (C, D) Correlations of insulin levels (C) and homeostasis model assessment of insulin resistance (HOMA-IR) (D) with serum IGF-1 levels in HCC patients. Linear regression coefficients and statistical significance are indicated.

To clarify the clinical significance of decreased IGF-1 levels in HCC, we determined whether serum IGF-1 levels correlated with prognosis. The median serum IGF-1 level of these 102 HCC patients was 44.8 ng/mL. On the basis of serum IGF-1 levels, we divided the whole study group into low (<44.8 ng/mL) and high serum IGF-1 (>44.8 ng/mL) level groups. Kaplan-Meier curves showed a significant association between low serum IGF-1 levels and poor overall survival (Figure 4A). However, the intergroup difference in the recurrence-free survival of patients was not statistically significant (Figure 4B). Further, the relationship between serum IGF-1 levels and clinicopathological features was examined. A significant inverse association was found between IGF-1 and insulin levels (Figure 4C), as well as between IGF-1 levels and insulin resistance index (HOMA-IR) (Figure 4D). However, there were no significant correlations between serum IGF-1 levels and tumor characteristics among HCC patients (Table S2). Taken together, these results suggest that low serum IGF-1 level is associated with insulin resistance and poor overall survival in HCC patients.

### Over-expression of miR-190b Contributes to Hepatic Insulin Resistance through Down-regulation of IGF-1 Expression

**Figure 5:**
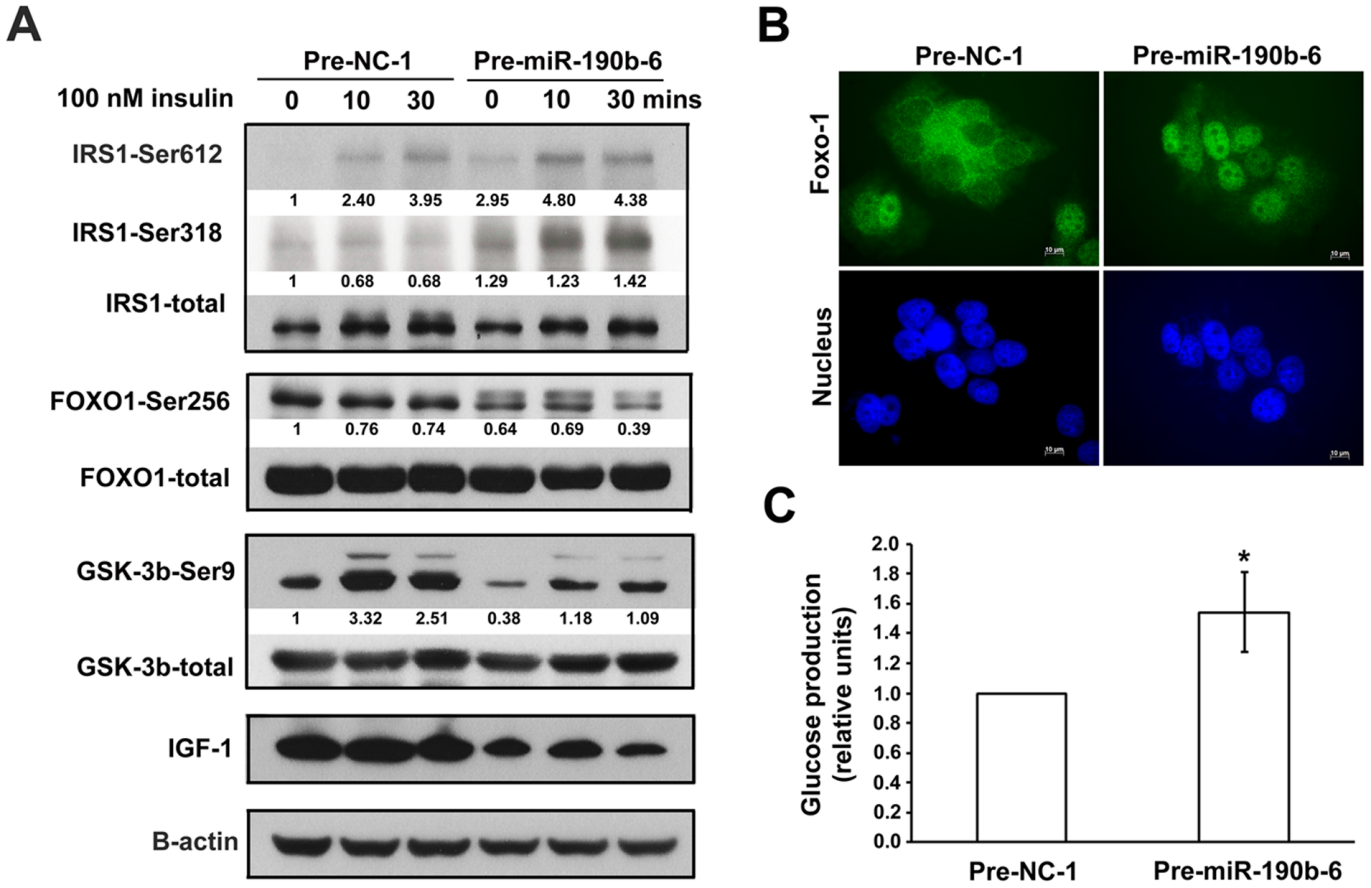
Over-expression of miR-190b in HCC cells leads to insulin resistance through down-regulation of IGF-1 expression. (A) Representative western blotting and quantification of expression and insulin-stimulated phosphorylation of IRS1, FOXO1 and GSK3β in Huh7 stable cells expressing precursor negative control (NC) or miR-190b. Cells were starved for 24 hours and then treated with 100 nM insulin for 10 and 30 minutes. Band intensity and ratio of phospho-form/total proteins were calculated by Image J. (B) immunofluorescence staining showing the subcellular localization of FOXO1 in Huh7 stable cells. (C) Extracellular glucose production in Huh7 stable cells was measured as described in Materials and Methods (*P<0.05).

As a potential regulator of IGF-1, we reasoned that expression of miR-190b might be involved in insulin resistance. To address this issue, we investigated the phosphorylation status of insulin signaling mediators by western blotting in the presence of miR-190b overexpression. The down-regulation of IGF-1 in Huh7-Pre-miR190b-6 cells was confirmed again in Figure 5A. Overexpression of miR-190b increased hepatic expression and insulin-stimulated phosphorylation of IRS1 (insulin receptor substrate-1) at Ser612 and Ser318 (Figure 5A), verifying impairment of insulin signaling [23]. Phosphorylation of forkhead box class O1 (FOXO1) and glycogen synthase kinase 3β (GSK3β), two downstream effectors of insulin action [24], revealed that overexpression of miR-190b critically decreased FOXO1 and GSK3β phosphorylation at Ser256 and Ser9, respectively (Figure 5A). In addition, upon treatment of insulin, miR-190b overexpression resulted in the diminished ability of insulin to phosphorylate GSK3β. Abnormalities in the signaling pathway, including IRS1, FOXO1 and GSK3β, marked the main characteristics of insulin resistance in Huh7-Pre-miR190b-6 cells.

The phosphorylated form of FOXO1, a transcription factor controlling gene expression of gluconeogenesis, is exported from the nucleus and thereby loses its transcriptional function [25]. Measurement of FOXO1 translocation and glucose production revealed that the majority of FOXO1 accumulated in the nucleus upon miR-190b overexpression, whereas in control cells, FOXO1 was distributed in both nucleus and cytoplasm (Figure 5B). Cells that overexpressed miR-190b produced greater amounts of glucose than did control cells (Figure 5C). Taken together, these results indicate the critical role of miR-190b in impairing insulin signaling and promoting hepatic gluconeogenesis in HCC cells.

### Restoration of IGF-1 Inhibits miR-190b-mediated Insulin Resistance

**Figure 6:**
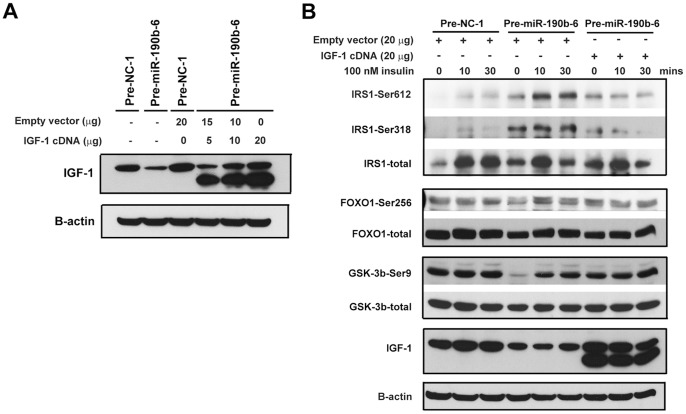
Restoration of IGF-1 inhibited miR-190b-mediated insulin resistance. (A) Western blotting of IGF-1 expression in Huh7-Pre-NC-1 cells or Huh7-Pre-miR190b-6 cells transfected with IGF-1 expression vector in different amounts. (B) Western blotting of expression and insulin-stimulated phosphorylation of IRS1, FOXO1 and GSK3β in Huh7-Pre-NC-1 cells transfected with empty vector, and Huh7-Pre-miR190b-6 cells transfected with empty vector or IGF-1 expression vector. β-actin was used as an internal control.

We next asked whether the insulin-resistance effects of miR-190b in HCC cells was attributed to its suppressive effect on IGF-1 expression. Transfection of Huh7-Pre-miR190b-6 cells with IGF-1 cDNA in a dose-dependent manner revealed that 20 microgram cDNA expresses comparable IGF-1 levels as control cells. A faster migrating band in IGF-1-overexpressing lysates may be IGF-1 isoform protein (Figure 6A).

Consistent with previously observed (Figure 5A), Huh7-Pre-miR190b-6 cells exhibited a significantly increased level of IRS1-Ser612&318 and decreased level of FOXO1-Ser256 and GSK3β-Ser9 when compared with control cells (Figure 6B). Restoring IGF-1 levels via cDNA transfection reversed the altered phophorylation status of Huh7-Pre-miR190b-6 cells to the similar level as control cells (Figure 6B). IGF-1 has been functionally validated as a *bona fide* target of miR-190b. In conclusion, the involvement of insulin resistance in response to IGF-1 deficiency was further substantiated by this *in* *vitro* evidence.

## Discussion

Since the decline of IGF-1 in HCC is not well understood, this study investigated whether dysregulation of a specific miRNA may modulate IGF-1 reduction, and if so, what the biological consequences may be. Results of this study have shown that HCC significantly increases expression of miR-190b, which targets to IGF-1 3′UTR directly, resulting in impaired insulin signaling and gluconeogenesis. These findings suggest a novel mechanism for the development of insulin resistance in HCC by providing the first evidence that miR-190b mediates the repression of IGF-1 expression.

Most circulating IGF-1 is produced by the liver and production is regulated by growth hormone (GH) [20]. IGF-1 deficiency in HCC is thought to result primarily from the reduced synthetic capacity of hepatic mass. Alternatively, it might be the result of reduced GH stimulation because GH receptor expression in hepatoma tissue is also low [26]. However, some studies report that reduction of IGF-1 among HCC patients is greater than that attributed to liver damage alone. Mazziotti et al. found that IGF-1 levels decreased independently from the progression of cirrhosis from Child Grade A to Child Grade B [18]. After controlling for the degree of liver damage, as assessed by prothrombin time and serum albumin level, Stuver et al. reported that reduction of serum IGF-1 levels in HCC patients appeared to be largely independent of liver damage [16]. Results of the present study have consistently shown that the gradually decreased IGF-1 levels among healthy subjects, viral hepatitis patients and HCC patients were partially independent of AST, ALT and total bilirubin levels. The idea that tumor-specific factors also contribute to the decline of IGF-1 in HCC was supported by evidence that overexpressed miR-190b in HCC tumor tissues regulated IGF-1 expression. Along the same line, some tumor cytokines, including Interleukin-1 beta, tumor necrosis factor-alpha and interleukin-6, were found to be elevated in HCC patients [27], have been reported to block IGF-1 production in the liver [28], [29].

IGF-1 gene is heavily epigenetically regulated. A study of intrauterine growth restriction (IUGR) in rats found that the modest extent of IGF-1 promoter DNA hypermethylation appears to dampen IGF-1 expression in the IUGR liver [30]. Another study of traumatic brain injury in rats demonstrated that increased hippocampal IGF-1B mRNA is associated with DNA methylation and/or histone modifications at the promoter site 1 or exon 5 regions [31]. Furthermore, it becomes increasingly clear that HCC initiation and promotion can occur by way of epigenetic mechanisms [32]. Therefore, effects of epigenetic modification on the down-regulation of IGF-1 expression in HCC might be worth future research.

The production levels of many metabolic factors, including insulin and IGF-1, are critically controlled at the post-transcriptional level. Post-transcriptional regulations that alter the stability and translation of encoding mRNA allow the cell to respond effectively to stimuli such as altered glucose levels [9], [33]. The liver is the principal organ that regulates glucose homeostasis because of its capacity to consume and produce glucose [34]. The liver’s central role in glucose homeostasis offers a clue to the hypothesis that reduction of IGF-1 in HCC is due to miRNA-mediated post-transcriptional regulation. Our finding that miR-190b has a regulatory role for IGF-1 expression in human HCC supports this hypothesis. Dysregulated miRNAs, which was identified by our experimental observations and those of other studies [13]–[15], indicate that defects in post-transcriptional regulation contribute to the pathogenesis of metabolic disorders at multiple levels, including impaired insulin signaling, gluconeogenesis, glucose-induced apoptosis and impaired angiogenesis.

The effect of IGF-1 on tumor formation in humans is demonstrated by the finding that patients with acromegaly with elevated serum IGF-1 levels exhibit increased risk of colon and thyroid cancer [35]. This increased level of IGF-1 is in keeping with its known mitogenic and anti-apoptotic effects. However, contrary to IGF-1 levels in other cancers, IGF-1 levels are decreased in HCC and the underlying mechanisms are difficult to explain based on the proliferative effect of IGF-1. We consistently found no significant correlations between serum IGF-1 levels and tumor characteristics among HCC patients (Table S2). Further, this study showed that serum IGF-1 levels can predict overall survival of HCC patients, but not recurrence-free survival (Figure 4A and B). Accordingly, we propose that the association of low serum IGF-1 levels with poor prognosis in HCC may be attributable to mechanisms beyond development of tumor itself.

In contrast to its negative effects on growth and proliferation, reduced IGF-1 signaling has been reported to positively correlated insulin resistance [3], [4]. Recently, many evidence points to a link between insulin resistance and cancer [36], [37]. In patients with chronic hepatitis C, HCC subjects have a higher insulin resistance index than those with chronic hepatitis [38]. Moreover, prognosis is worse in HCC patients with glucose intolerance or increased fasting serum insulin level [39], [40]. In accordance with these lines of evidence, low serum IGF-1 levels were associated with insulin resistance and poor prognosis in our HCC patients. Besides, we showed that the impaired insulin signaling caused by overexpression of miR-190b in HCC cells can partly reverse by restoring IGF-1 expression. These data indicated decreased expression of IGF-1 have a role in the development of HCC-associated insulin resistance. For the first time, this study demonstrated that insulin resistance appears to be part of the physiopathologic significance of decreased IGF-1 levels in HCC progression.

Recently, an association between IGF-1 and HCC patient outcome had been reported in the literature [41], [42]. Shao et al. reported that high pretreatment IGF-1 level were associated with better survival and disease control rate of patients who received antiangiogenic therapy for advanced HCC [41]. Because of the lack of a control group not receiving therapy, it was uncertain that the better survival outcomes of high IGF-1 group resulted from better tumor prognosis or better therapeutic efficacy, or both. Kaseb et al. reported that low plasma IGF-1 levels correlated with poor overall survival in the other HCC patient cohort with heterogeneous disease status and treatment [42]. The main difference between our and the previous studies is that all patients in this study received curative resection, which resulted in a more homogeneous patient population. Moreover, after resection of HCC, none of the patients had received adjuvant therapy. Thus, we can conclude that the better survival outcomes of patients with high preoperative serum IGF-1 were derived from better tumor prognosis. In this study, we were able to substantiate earlier observations and extended our investigations to determine the biological role of IGF-1 in HCC.

As miR-190b was indentified between HCC tissues and their paired non-tumor tissues, we wondered whether miR-190b has potential effects on the characteristics of HCC. We checked the relationship of miR-190b with overall survival and found that high miR-190b levels could infer a poorer overall survival prospect of HCC patients (Figure S3-A). However, the intergroup difference was not statistically significant, which may be beset with small sample-size (n = 29). Further, the relationship between miR-190b levels and clinicopathological features was examined. However, there were no significant correlations between miR-190b levels and tumor characteristics among HCC patients (Table S3). Additional studies with large sample sizes are required to confirm these results.

Besides HCC, up-regulation of miR-190b has been reported in miRNA profile studies of other cancers such as rectal cancer and lung cancer [43], [44]. However, in none of these reports has the biological consequences of miR-190b dysregulation in human cancers been characterized further. We examined whether the miR-190b overexpression would affect the cancer biology. The WST-1 assay demonstrated a modest, but significant, decrease in cell viability upon miR-190b overexpression (Figure S3-B). In general, slowly proliferating cells are far more resistant to chemotherapeutic drug treatment [45]. Therefore, an *in* *vitro* chemosensitivity assay was then performed. We observed that the overexpression of miR-190b increased resistance to 5-Fluorouracil (Figure S3-C). These results are in line with a previous report showing the up-regulation of miR-190b in rectal tumors of non-reponders to chemoradiotherapy [43]. Next, we evaluated the apoptosis potential of miR-190b by caspase 3/7 activity assay. As shown in Figure S3-D, increased expression of miR-190b inhibited cell apoptosis in HCC cells. Given that IGF-1 is the main anti-apoptoic factor, we supposed that additional mechanisms induced by miR-190b were involved in this anti-apoptosis ability, independently of IGF-1 inhibition. Our present study substantiates the notion that a single miRNA could pleiotropically regulate several oncogenic targets.

In summary, the present study not only uncovered that post-transcriptional regulation is responsible for the decline of IGF-1, but also elucidated the mechanisms by which IGF-1 affects the clinical course of HCC patients. The therapeutic strategy that supplementation of IGF-1 to improve metabolic control after curative resection for HCC may be worth further study. Furthermore, the findings that the dysregulation of miR-190b had effects on drug resistance and anti-apoptosis indicate that miR-190b could be a therapeutic target of HCC.

## Supporting Information

Figure S1 **miR-190b repressed IGF-1** **mRNA stability.**
(PDF)

Figure S2 **miR-190b regulated IGF-1 protein expression.** Huh7 cells were transiently transfected with pre-NC, pre-miR-190b, anti-NC or anti-miR-190b vectors.
(PDF)

Figure S3 **miR-190b overexpression was correlated with prognosis in HCC patients and regulated cell viability, chemosensitivity and apoptosis in HCC cells.**
(PDF)

Table S1 **List of 16 significantly dysregulated microRNAs in human hepatocelluar carcinoma (HCC) (miRNA beadarray data).**
(DOC)

Table S2 **Comparison of tumor characteristics between low and high serum insulin-like growth factor (IGF)-1 levels in patients with hepatocellular carcinoma.**
(DOC)

Table S3 **Comparison of tumor characteristics between low and high miR-190b levels in patients with hepatocellular carcinoma.**
(DOC)
