# Supplementary material for: Up-Regulation of MicroRNA-190b Plays a Role for Decreased IGF-1 That Induces Insulin Resistance in Human Hepatocellular Carcinoma
Source: PLoS One. 2014 Feb 20;9(2):e89446. doi: 10.1371/journal.pone.0089446 (PMC3930738; doi:10.1371/journal.pone.0089446)
Supplement: Figure S1 — miR-190b repressed IGF-1 mRNA stability. (PDF) [file pone.0089446.s001.pdf]

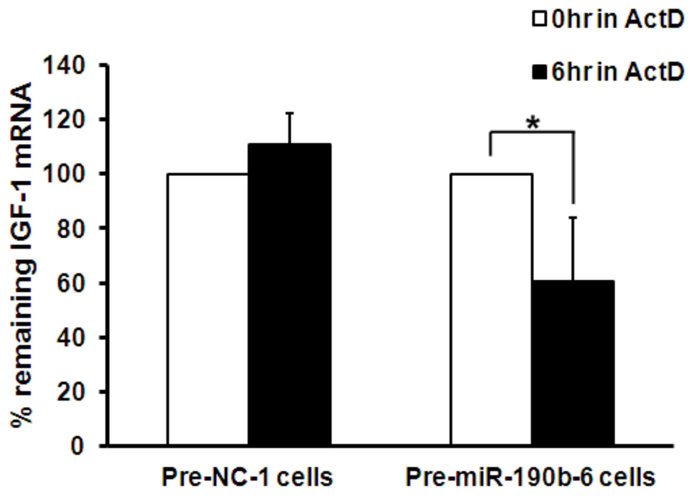

Figure S1. miR-190b repressed IGF-1 mRNA stability. Huh7 cells stably expressing precursor negative control (Pre-NC) or precursor miR-190b (Pre-miR-190b) were treated with or without actinomycin D (ActD). RNA was isolated; subjected to qRT-PCR analysis to measure the levels of IGF-1 mRNA. mRNA stability was assessed by comparing the amount remaining after culture with ActD with the initial level without ActD. IGF-1 mRNA is more rapidly degraded in miR-190b overexpressed cells than control cells, and with only 60% remaining after 6 hours in the presence of ActD in miR-190b cells. The error bars indicate standard deviations of 3 independent experiments. * $P < 0.05$
