# Supplementary material for: Up-Regulation of MicroRNA-190b Plays a Role for Decreased IGF-1 That Induces Insulin Resistance in Human Hepatocellular Carcinoma
Source: PLoS One. 2014 Feb 20;9(2):e89446. doi: 10.1371/journal.pone.0089446 (PMC3930738; doi:10.1371/journal.pone.0089446)
Supplement: Figure S2 — miR-190b regulated IGF-1 protein expression. Huh7 cells were transiently transfected with pre-NC, pre-miR-190b, anti-NC or anti-miR-190b vectors. (PDF) [file pone.0089446.s002.pdf]

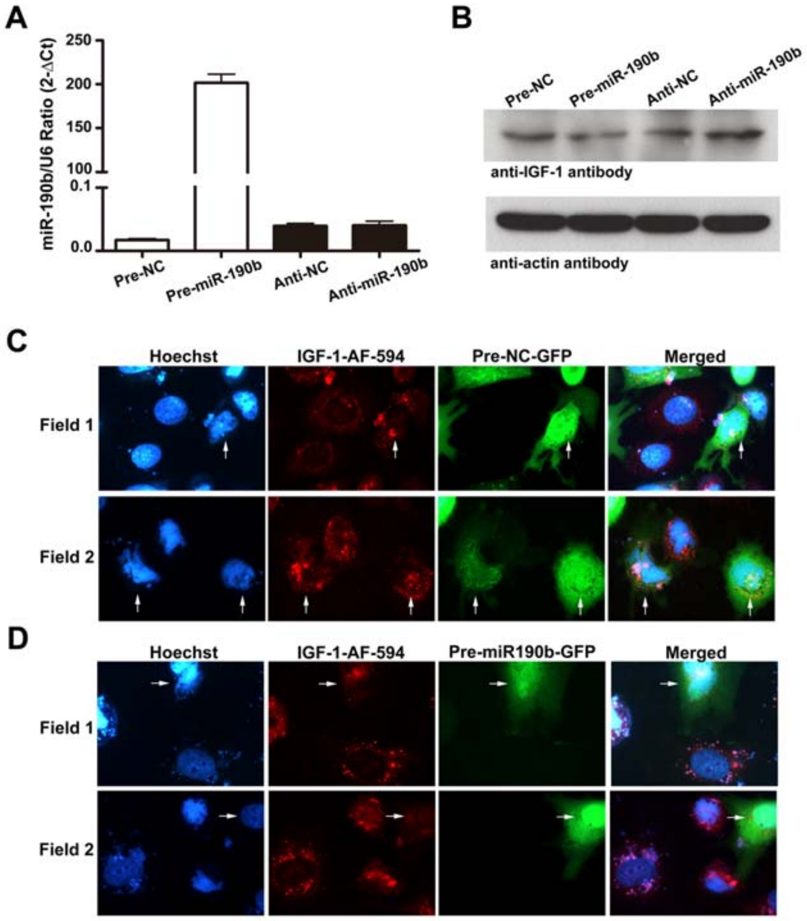

Figure S2. miR-190b regulated IGF-1 protein expression. Huh7 cells were transiently transfected with pre-NC, pre-miR-190b, anti-NC or anti-miR-190b vectors. **(A)** Expression of miR-190b in Huh7 transfectant was confirmed by qRT-PCR and normalized to the levels of U6. **(B)** Overexpression of miR-190b attenuated IGF-1 protein expression, and blocking miR-190b increased IGF-1 protein expression. **(C and D)** Immunofluorescent images of Huh7 transfectants. Cells were transiently transfected with empty vector (C) or vector expressing miR-190b (D). Two days after transfection, cells were fixed with 4% paraformaldehyde and subjected to immunofluorescence staining. Successful transfected cells in each field are green due to EGFP fluorescence. IGF-1 (red fluorescence) was detected using rabbit anti-IGF-1 antibodies and Alexa Fluor-594 (AF-594)-conjugated goat anti-rabbit immunoglobulin G. Hoechst 33258 was used along with the secondary antibody to detect the nucleus. IGF-1 expression was suppressed in the cells transfected with precursor miR-190b (arrow in D). Cells transfected with empty vector showed no change in IGF-1 expression (arrow in C).
