# Supplementary material for: Up-Regulation of MicroRNA-190b Plays a Role for Decreased IGF-1 That Induces Insulin Resistance in Human Hepatocellular Carcinoma
Source: PLoS One. 2014 Feb 20;9(2):e89446. doi: 10.1371/journal.pone.0089446 (PMC3930738; doi:10.1371/journal.pone.0089446)
Supplement: Figure S3 — miR-190b overexpression was correlated with prognosis in HCC patients and regulated cell viability, chemosensitivity and apoptosis in HCC cells. (PDF) [file pone.0089446.s003.pdf]

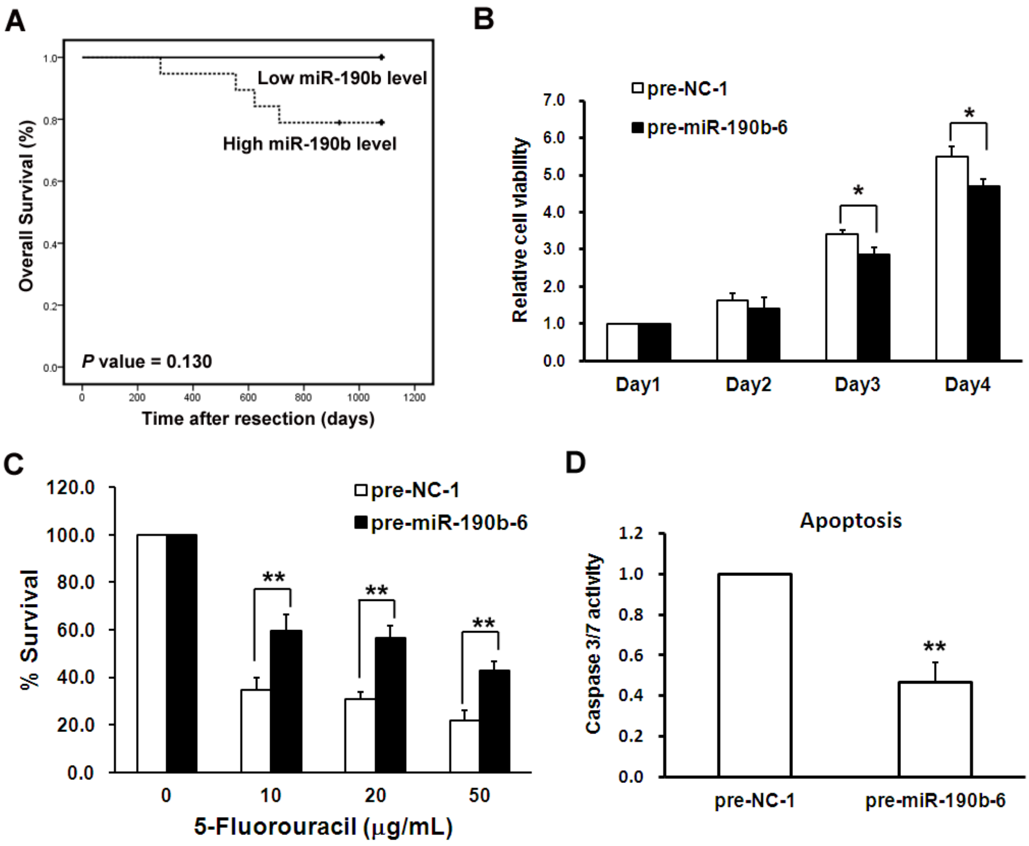

Figure S3. miR-190b overexpression was correlated with prognosis in HCC patients and regulated cell viability, chemosensitivity and apoptosis in HCC cells. (A) Kaplan-Meier curves for overall survival in 29 HCC patients. HCC patients were divided into two groups according to whether or not the miR-190b levels were up-regulated more than 2-fold in tumor tissue (T) when compared with paired non-tumor tissue (NT). 'Low' denotes T/NT ratio <2 (n=10), and 'High' denotes T/NT ratio >=2 (n=19). Statistical significance was calculated using the log-rank test. **(B) Effect of miR-190b on cell proliferation was assessed by WST-1 assay.** Cells were seeded in triplicate in 96-well plates. At the time of harvest, cells were treated with WST-1 reagent (Roche) and incubated in 37°C for one hour. Cell viability was determined by measuring the absorbance of the converted dye at 450 nm. Huh7 cells stably expressing precursor miR-190b (Pre-miR-190b-6) exhibited a reduced proliferation when compared with negative control cells (named Pre-NC-1). **(C)**

Chemosensitivity to 5-Fluorouracil (FU) of Huh7-Pre-miR-190b-6 and Huh7-Pre-NC-1 cells. Cells were seeded in triplicate in 96-well plates. After 24 hours, medium containing FU were added, and the cells were cultured for an additional 48 hours. At the time of harvest, cell viability was measured by WST-1 assay as described earlier. The results were expressed as the percentage of the difference between untreated and treated cells. **(D) Effect of miR-190b on cell apoptosis was assessed by caspase 3/7 activity assay.** Cells were seeded in triplicate in 96-well plates. After 24 hours, serum free medium were added, and the cells were cultured for an additional 48 hours. At the time of harvest, caspase 3 and 7 activities were measured using a Caspase-Glo assay kit (Promega) according to the manufacturer’s instructions. Data expressed as the mean ± SD correspond to three separate experiments. * $P < 0.05$; ** $P < 0.01$
