# Supplementary material for: Up-Regulation of MicroRNA-190b Plays a Role for Decreased IGF-1 That Induces Insulin Resistance in Human Hepatocellular Carcinoma
Source: PLoS One. 2014 Feb 20;9(2):e89446. doi: 10.1371/journal.pone.0089446 (PMC3930738; doi:10.1371/journal.pone.0089446)
Supplement: Table S1 — List of 16 significantly dysregulated microRNAs in human hepatocelluar carcinoma (HCC) (miRNA beadarray data). (DOC) [file pone.0089446.s004.doc]

**Table S1.** List of 16 significantly dysregulated microRNAs in human hepatocelluar carcinoma (HCC) (miRNA beadarray data).

|  | **miRNA** | **Mean intensity**  **in NT*** | **Mean intensity**  **in T*** | **Ratio**  **(T/NT)** | ***P* value** |
| --- | --- | --- | --- | --- | --- |
| **Up-regulated in HCC** | | | | | |
| 1 | miR-190b | 730 ± 191 | 1619 ± 1315 | 2.22 | 0.00001 |
| 2 | miR-182 | 2352 ± 1148 | 9587 ± 7534 | 4.08 | 0.00002 |
| 3 | miR-183 | 1639 ± 890 | 8080 ± 8174 | 4.93 | 0.00005 |
| 4 | miR-10b | 1244 ± 548 | 3839 ± 4043 | 3.09 | 0.00023 |
| 5 | miR-501-5p | 1214 ± 608 | 2771 ± 2495 | 2.28 | 0.00044 |
| 6 | miR-452 | 1750 ± 910 | 4420 ± 4028 | 2.53 | 0.00071 |
| 7 | miR-96 | 951 ± 348 | 2149 ± 1786 | 2.26 | 0.00090 |
| 8 | miR-196b | 1413 ± 169 | 3252 ± 3517 | 2.30 | 0.00295 |
| **Down-regulated in HCC** | | | | | |
| 9 | miR-214* | 5208 ± 1990 | 1836 ± 1118 | 0.35 | 0.00001 |
| 10 | miR-199a-5p | 33338 ± 3442 | 16520 ± 11102 | 0.50 | 0.00001 |
| 11 | HS_50 | 1189 ± 856 | 607 ± 136 | 0.50 | 0.00001 |
| 12 | miR-139-3p | 2721 ± 872 | 1362 ± 657 | 0.50 | 0.00002 |
| 13 | miR-424* | 4518 ± 1388 | 2254 ± 1404 | 0.50 | 0.00003 |
| 14 | miR-200b* | 5721 ± 2196 | 2427 ± 2483 | 0.42 | 0.00006 |
| 15 | miR-200a | 8412 ± 4708 | 3845±4963 | 0.46 | 0.00019 |
| 16 | miR-375 | 16896 ± 7852 | 7111 ± 8465 | 0.42 | 0.00071 |

* The expression of microRNAs in tumor (T) and paired non-tumor (NT) tissues are presented as mean ± SD and analyzed using Wilcoxon signed rank test.
