# Supplementary material for: Up-Regulation of MicroRNA-190b Plays a Role for Decreased IGF-1 That Induces Insulin Resistance in Human Hepatocellular Carcinoma
Source: PLoS One. 2014 Feb 20;9(2):e89446. doi: 10.1371/journal.pone.0089446 (PMC3930738; doi:10.1371/journal.pone.0089446)
Supplement: Table S2 — Comparison of tumor characteristics between low and high serum insulin-like growth factor (IGF)-1 levels in patients with hepatocellular carcinoma. (DOC) [file pone.0089446.s005.doc]

**Table S2** Comparison of tumor characteristics between low and high serum insulin-like growth factor (IGF)-1 levels in patients with hepatocellular carcinoma

|  |  | Serum IGF-1 level | |  |
| --- | --- | --- | --- | --- |
|  | Patients | Low | High | *P* value |
| Feature | n^#^ | n (%) | n (%) |  |
| All cases | 102 | 51 | 51 |  |
| Age (years) | 102 | 59.6 ± 11.2 | 56.6 ± 11.8 | 0.183^a^ |
| Gender |  |  |  | 0.250^b^ |
| Male | 77 | 36 (46.8) | 41 (53.2) |  |
| Female | 25 | 15 (60.0) | 10 (40.0) |  |
| Serum AFP level (ng/mL) | 84 | 53.9 (2.5–87500) | 20.0 (1.5–87500) | 0.053^c^ |
| Histologic grade |  |  |  | 0.790^d^ |
| I: well differentiated | 3 | 1 (33.3) | 2 (66.7) |  |
| II: moderately differentiated | 54 | 29 (53.7) | 25 (46.3) |  |
| III: poorly differentiated | 28 | 15 (53.6) | 13 (46.4) |  |
| IV: undifferentiated | 1 | 0 (0.0) | 1 (100) |  |
| TNM stage |  |  |  |  |
| I | 53 | 23 (43.4) | 30 (56.6) | 0.084^b^ |
| II | 29 | 14 (48.3) | 15 (51.7) |  |
| III | 16 | 12 (75.0) | 4 (25.0) |  |
| Tumor size |  |  |  | 0.068^b^ |
| < 5 cm | 62 | 27 (43.5) | 35 (54.5) |  |
| ≥ 5 cm | 35 | 22 (62.9) | 13 (37.1) |  |
| Vascular invasion |  |  |  | 0.300^b^ |
| Absent | 63 | 29 (46.0) | 34 (54.0) |  |
| Present | 37 | 21 (56.8) | 16 (43.2) |  |
| Intrahepatic recurrence |  |  |  | 0.413^b^ |
| Absent | 38 | 21 (55.3) | 17 (44.7) |  |
| Present | 64 | 30 (46.9) | 34 (53.1) |  |
| Extrahepatic metastasis |  |  |  | 0.603^b^ |
| Absent | 84 | 41 (48.8) | 43 (51.2) |  |
| Present | 18 | 10 (55.6) | 8 (44.4) |  |

Significant *P* values are in bold.

^#^The total number of patients was less than 102 because some data were missing.

^a^Student’s *t* test; ^b^ Chi-square test; ^c^Mann-Whitney U test; ^d^ Fisher’s exact test.

Age are expressed as mean ± SD, and statistical analyses was performed using Student’s *t* test.

Serum AFP levels are expressed as median and range, and statistical analyses was performed using Mann-Whitney U test.

AFP, alpha-fetoprotein
