# Supplementary material for: Up-Regulation of MicroRNA-190b Plays a Role for Decreased IGF-1 That Induces Insulin Resistance in Human Hepatocellular Carcinoma
Source: PLoS One. 2014 Feb 20;9(2):e89446. doi: 10.1371/journal.pone.0089446 (PMC3930738; doi:10.1371/journal.pone.0089446)
Supplement: Table S3 — Comparison of tumor characteristics between low and high miR-190b levels in patients with hepatocellular carcinoma. (DOC) [file pone.0089446.s006.doc]

**Table S3** Comparison of tumor characteristics between low and high miR-190b levels in patients with hepatocellular carcinoma

|  |  | miR-190b expression* | |  |
| --- | --- | --- | --- | --- |
|  | Patients | Low | High | *P* value |
| Feature | n^#^ | n (%) | n (%) |  |
| All cases | 29 | 10 (34.5) | 19 (65.5) |  |
| Age (years) | 29 | 60.6 ± 8.6 | 62.2 ± 10.9 | 0.691^a^ |
| Gender |  |  |  | 1.000^b^ |
| Male | 22 | 8 (36.4) | 14 (63.6) |  |
| Female | 7 | 2 (28.6) | 5 (71.4) |  |
| Virus Status |  |  |  | 0.919^b^ |
| HBV | 13 | 4 (30.8) | 9 (69.2) |  |
| HCV | 10 | 4 (40.0) | 6 (60.0) |  |
| Non-B, Non C | 6 | 2 (33.3) | 4 (66.7) |  |
| Alcohol |  |  |  | 0.669^b^ |
| Yes | 7 | 2 (28.6) | 5 (71.4) |  |
| No | 17 | 7 (41.2) | 10 (58.8) |  |
| Cirrhosis |  |  |  | 0.449^b^ |
| Absent | 12 | 3 (25.0) | 9 (75.0) |  |
| Present | 17 | 7 (41.2) | 10 (58.8) |  |
| Serum AFP level (ng/mL) | 26 | 7.3 (2.8–10908) | 15.7 (2.3–3000.6) | 0.752^c^ |
| Histologic grade |  |  |  | 0.153^b^ |
| I: well differentiated | 3 | 2 (66.7) | 1 (33.3) |  |
| II: moderately differentiated | 16 | 5 (31.3) | 11 (68.7) |  |
| III: poorly differentiated | 8 | 1 (12.5) | 7 (87.5) |  |
| IV: undifferentiated | 1 | 1 (100) | 0 (0.0) |  |
| TNM stage |  |  |  |  |
| I | 19 | 8 (42.1) | 11 (57.9) | 0.619^b^ |
| II | 6 | 1 (16.7) | 5 (83.3) |  |
| III | 4 | 1 (25.0) | 3 (75.0) |  |
| Tumor size |  |  |  | 0.306^b^ |
| < 5 cm | 24 | 7 (29.2) | 17 (70.8) |  |
| ≥ 5 cm | 5 | 3 (60.0) | 2 (40.0) |  |
| Vascular invasion |  |  |  | 0.633^b^ |
| Absent | 24 | 9 (37.5) | 15 (62.5) |  |
| Present | 5 | 1 (20.0) | 4 (80.0) |  |
| Intrahepatic recurrence |  |  |  | 0.433^b^ |
| Absent | 13 | 3 (23.1) | 10 (76.9) |  |
| Present | 16 | 7 (43.8) | 9 (56.2) |  |
| Extrahepatic metastasis |  |  |  | 0.665^b^ |
| Absent | 22 | 7 (31.8) | 15 (68.2) |  |
| Present | 7 | 3 (42.9) | 4 (57.1) |  |

^#^The total number of patients was less than 29 because some data were missing.

*miR-190b expression that up-regulated more than 2-fold in tumor tissues when compared with paired non-tumor tissues denotes as “High” group

^a^Student’s *t* test; ^b^ Fisher’s exact test; ^c^Mann-Whitney U test

Age are expressed as mean ± SD, and statistical analyses was performed using Student’s *t* test.

Serum AFP levels are expressed as median and range, and statistical analyses was performed using Mann-Whitney U test.

AFP, alpha-fetoprotein
